# Loop-Mediated Isothermal Amplification in Schistosomiasis

**DOI:** 10.3390/jcm10030511

**Published:** 2021-02-01

**Authors:** Juan García-Bernalt Diego, Pedro Fernández-Soto, Begoña Febrer-Sendra, Beatriz Crego-Vicente, Antonio Muro

**Affiliations:** Infectious and Tropical Diseases Research Group (e-INTRO), Biomedical Research Institute of Salamanca-Research Centre for Tropical Diseases at the University of Salamanca (IBSAL-CIETUS), Faculty of Pharmacy, University of Salamanca, 37007 Salamanca, Spain; juanbernalt95@usal.es (J.G.-B.D.); begofebrer@usal.es (B.F.-S.); beatrizcregovic@usal.es (B.C.-V.); ama@usal.es (A.M.)

**Keywords:** schistosomiasis, LAMP, diagnosis, point-of-care, neglected tropical diseases, molecular diagnostics

## Abstract

Human schistosomiasis is one of the most important parasitic diseases, causing around 250 million cases (mostly in Africa) and 280,000–500,000 deaths every year. Due to the limited resources and the far-removed nature of many endemic areas, the implementation of new, sensitive and specific diagnostic tools has had little success. This is particularly true for PCR-based molecular methods that require expensive equipment and trained personnel to be executed. Loop-mediated isothermal amplification (LAMP) along with other isothermal techniques appeared in the early 21st century as an alternative to those methods, overcoming some of the aforementioned limitations and achieving a more inexpensive diagnostic. However, to this date, neither LAMP nor any other isothermal technique have signified a meaningful change in the way schistosomiasis diagnosis is routinely performed. Here, we present the recent developments in LAMP-based schistosomiasis diagnosis. We expose the main advantages and disadvantages of LAMP technology over PCR and other classical diagnostic methods focusing in various research approaches on intermediate hosts, animal models and patients. We also examine its potential clinical application in post-therapy monitoring, as well as its usefulness as a point-of-care test.

## 1. Introduction

Human schistosomiasis is the most important helminthic Neglected Tropical Disease (NTD), causing significant morbidity and mortality [1]. It is a chronic waterborne parasitic disease caused by several flatworm (blood flukes) trematodes of the genus *Schistosoma*. It is transmitted in 78 countries with over 250 million estimated cases (mostly in Africa), around 280,000–500,000 deaths every year, and a DALYs index of 3.3 million per year [2]. The three most widespread and clinically impactful *Schistosoma* species affecting humans are *Schistosoma haematobium* (Africa and the Middle East), *S. mansoni* (Africa, South America, the Middle East and the Caribbean), and *S. japonicum* (Indonesia, China, Southeast Asia and the Philippines). Moreover, there are four additional *Schistosoma* species able to infect humans, which present a lower prevalence: *S. mekongi*, *S. guineensis*, *S. intercalatum* and *S. malayensis* [3]. Additionally, the hybridization between *Schistosoma* species is an increasing concern [4]. Schistosomiasis is acquired by contact with freshwater contaminated with cercariae penetrating the skin that are disseminated by specific intermediate-host snails [1,3]. Transmission typically occurs in tropical and subtropical regions (80–90% of cases in sub-Saharan Africa). Nevertheless, recent outbreaks caused by schistosome hybrids in the south of Europe have proved the reemergence potential of the disease in temperate regions [5,6]. Clinically, three stages are typically distinguished: cercarial dermatitis, acute and chronic schistosomiasis. *Cercarial dermatitis* is due to skin penetration of cercariae, which cause a maculopapular pruritic reaction that lasts for 24–48 h. This is notably more common among migrants or travelers than residents in endemic areas [7]. *Acute schistosomiasis* (Katayama fever) usually occurs 2–4 weeks after infection, mostly in naive individuals, with rapid fever, fatigue, myalgia, malaise and eosinophilia, a consequence of a hypersensitivity reaction to the migrating schistosomulum. Rarely observed in people in schistosomes-endemic areas [8]. Finally, *chronic schistosomiasis* (months or years after infection) results from the granulomatous reaction around eggs trapped in the tissues [1]. “Classic” manifestations are species-dependent, being mainly intestinal and hepatic symptoms (*S. mansoni*, *S. japonicum*, *S. mekongi and S. intercalatum*) except in *S. haematobium* infections, which cause urogenital symptoms [1,2,9]. Additionally, *S. haematobium* chronic infection has been strongly correlated with bladder squamous cell carcinoma [10]. Ectopic lesions can also occur, and organs affected by ectopic granulomas include the central nervous system, genital organs, skin and eyes [11].

Schistosomiasis diagnosis requires different methods in acute and chronic infections. An active *Schistosoma* infection is definitively diagnosed by microscopic examination of excreted eggs in stool (*S. mansoni, S. japonicum, S. intercalum, S. guineensis* and *S. mekongi*) by Kato-Katz thick smear (KK), or in urine (*S. haematobium*) by filtration or sedimentation techniques. Acute infections, particularly those of low intensity, are frequently missed by microscopy [1,3]. A large number of serological approaches detecting antigens or antibodies have been widely evaluated in endemic areas, for imported or travel-related schistosomiasis, for epidemiological studies and control programs. However, significant differences in sensitivity and specificity exist, aggravated by a lack of standardization [12,13]. Numerous PCR-based assays have also been employed both in the diagnosis of animal and human schistosomiasis [14], being especially valuable in simultaneous detection and identification of *Schistosoma* species [15]. Despite being highly sensitive and accurate, the complex PCR-based methods for schistosomiasis are more difficult to adapt to field studies. In this context, isothermal nucleic acid amplification tests (iNAAT), particularly LAMP technology, much more suited for limited-resource settings, have represented a very promising alternative. Here, we present an overview highlighting the most relevant research performed with LAMP technology for schistosomiasis diagnosis. We discussed its application in different fields, from epidemiological surveys to clinical practice, including studies in intermediate hosts, in animal models and in humans, as well as its potential as a tool to assess schistosomiasis treatment success. We also discuss its role in the development of true point-of-care diagnostics for schistosomiasis, exposing both its advantages and disadvantages and pointing out the steps that are still needed to move forward.

## 2. Loop-Mediated Isothermal Amplification (LAMP)

LAMP technique was first introduced by Notomi et al. in 2000 [16] and, to date, it is the most widely used iNAAT among alternatives to PCR-based technologies. Briefly, LAMP assay is a one-step amplification reaction that amplifies a target DNA or RNA under isothermal conditions (60–65 °C) employing a DNA polymerase (*Bst* polymerase) with strand-displacement activity, along with two inner primers (FIP, BIP; forward and backward inner primers) and two outer primers (F3, B3; forward and backward outer primers) which specifically recognize six separate regions within the target. Shortly after LAMP description, Nagamine et al. [17] reported a major improvement by adding extra LAMP loop primers (LF, LB; loop-forward and loop-backward primers), thus shortening the reaction time by around 30 min. Hence, a six-primer design can be used in LAMP reactions, targeting in up to eight different regions, compared to only two in typically PCR-based methodology. Another important feature of LAMP is the numerous approaches towards result detection, including gel electrophoresis with characteristic amplicon ladder-like pattern [16], naked-eye monitoring of turbidity caused by the precipitation of magnesium pyrophosphate [18,19] as well as end-point detection by the addition of colorimetric dyes, either post-amplification [20] or pre-amplification [21,22,23,24]. The latest advances in end-point detection and in real-time monitoring of LAMP have been recently reviewed by Zhang et al. [25]. Compared to other molecular techniques, the simplicity of LAMP makes it ideal for field-testing in developing countries [18,21]. In recent years, a great variety of approaches have been used to further ensure that LAMP becomes a field-friendly tool, including lateral flow dipsticks and lab-on-chip layouts [26], microfluidic-based methods [27], combination with metallic nanoparticles [28], control through smart phone-based technology [29] and within a red blood cell [30].

## 3. LAMP and Schistosomiasis

**Figure 1:**
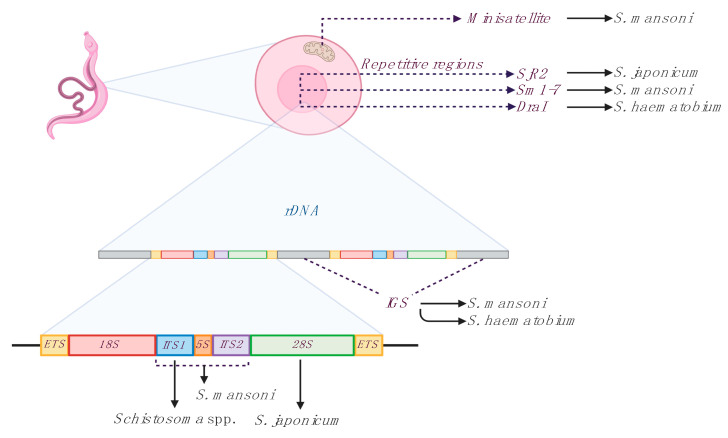
Main targets used for loop-mediated isothermal amplification (LAMP) designing for detection of *Schistosoma* species. Three groups of sequences are the most used for primer sets designing: mithocondrial sequences, repetitive nuclear sequences and ribosomal nuclear sequences (rDNA). Minisatellite; mithocondrial minisatellite sequence. Repetitive regions; SjR2, *S. japonicum* non-long terminal repeat retrotransposon; Sm1–7, *S. mansoni* 121 bp tandemly arranged repeated sequence; DraI, *S. haematobium* 121 bp tandemly arranged repeated sequence. Ribosomal nuclear sequences (rDNA); IGS, intergenic spacer sequences; ITS1 and ITS2, internal transcribed spacers; 5S, small subunit of ribosomal RNA; 18S, 28S, major subunits of ribosomal RNA; ETS, external transcribed spacers. Sm1–7 and DraI targets are here represented as species-specific, although they are considered more group-specific [32]. Figure created with BioRender software (https://biorender.com/).

A number of LAMP assays have been designed for the species-specific detection of the three main species causing human schistosomiasis (*S. haematobium*, *S. japonicum* and *S. mansoni*) and have been applied to schistosomiasis diagnosis, to detect schistosomes-infected snails, and to evaluate efficacy of chemotherapy, both in animal models and human patients. Recently, a LAMP for species-specific detection of the most important veterinary species (*S. bovis*) and a LAMP for simultaneous detection of different *Schistosoma* species have been also reported [31]. The selected molecular targets mostly used for LAMP designing for the detection of *Schistosoma* species are shown in Figure 1, and different assays features are summarized in Table 1.

### 3.1. LAMP in Schistosomes Infected Snails

To evaluate the efficiency of LAMP detecting schistosomes-infected snails, several experimental infections of snails of the genus *Oncomelania* (*S. japonicum*) [33], *Bulinus* (*S. haematobium*) [34] and *Biomphalaria* (*S. mansoni*) [34,35] have been carried out. In all cases, LAMP could detect schistosomes DNA during the early prepatent phase of infection (as soon as one day after exposure to one miracidium), ergo, before cercarial release, in both individual snails and pooled samples (up to 1 infected snail within 1000 non-infected snails [36]. LAMP has also been evaluated for the detection of *S. japonicum*, *S. haematobium* and *S. mansoni* in large-scale screening of pooled field-collected snails to evaluate schistosomiasis transmission, especially in low-prevalence areas [36,37,38,39,40]. In general, the studies performed in the intermediate host reveal that LAMP can be used as a rapid, sensitive, and inexpensive tool to screen large numbers of intermediate hosts compared to other molecular methods. Moreover, the usefulness of the tool to indentify transmission foci and build infection-risk maps has been shown for both *S. mansoni* [39] and *S. japonicum* [33,36]. Thus, the application of the technique could support schistosomiasis control campaigns.

### 3.2. LAMP in Schistosomes Experimental Infections

Animal models have profoundly contributed to the design and evaluation of LAMP assays for schistosomiasis, particularly for *S. mansoni* and *S. japonicum*. They have allowed the testing of the performance of the assays in different specimens as well as the sensitivity of the diagnosis along the course of the infection. In the case of *S. mansoni*, LAMP has been used to detect cell-free DNA (cfDNA) in infected mice in serum, plasma [41], stool samples [42,43] and urine [44]. For *S. japonicum*, cfDNA has been detected in stool, serum [45,46] and blood samples [47], obtained from infected rabbits. For both species, cfDNA from the parasite was detected in the acute phase of the infection, before Schistosoma eggs were detected in faeces [41,42,45] and even before immunological [42,46] or molecular [47] methods. Therefore, LAMP has shown a high potential as an early-detection diagnostic tool, as well as a sensitive diagnostic method for active infections.

**Table 1:** LAMP assays for *Schistosoma* species detection and their clinical application.

	Assay Features				Clinical Application				
Species ^1^	Target ^2^	Detection ^3^	Sensitivity ^4^	Specimen ^5^	*n* ^6^	Sensitivity (LAMP+/Ref+)	Specificity (LAMP−/Ref−)	Ref. Diag ^7^	Ref.
*S* spp.	*ITS-1*	SGI/Gel	0.1 pg *Sh* 1 pg *Sm/Si* 10 pg *Sb*	gDNA	N/A	N/A	N/A	N/A	Fernández-Soto et al. [31]
*Sh*	*DraI*	SGI/Gel	0.1 fg	gDNA, snails	N/A	N/A	N/A	N/A	Abbasi et al. [34]
	*DraI*	SGI/Gel	N/D	Snails	N/A	N/A	N/A	N/A	Hamburguer et al. [37]
	*IGS*	SGI/Gel	1 fg	gDNA, Urine	94	100% (18/18)	86.7% (68/76)	Micro	Gandasegui et al. [48]
	*DraI*	SGI/Gel	N/D	Urine	86	100% (72/72)	100% (14/14)	PCR	Lodh et al. [49]
	*IGS*	T/SGI/Gel	N/D	Urine	172	86.2% (75/87)	N/D	Micro	Gandasegui et al. [50]
*Sm*	*Sm1–7*	SGI/Gel	0.1 fg	gDNA, Snails	N/A	N/A	N/A	N/A	Abbasi et al. [34]
	*Sm1–7*	SGI/Gel	N/D	Snails	N/A	N/A	N/A	N/A	Hamburguer et al. [37]
	*Mito*	SGI/Gel	1 fg	gDNA, mice stool	N/A	N/A	N/A	N/A	Fernández-Soto et al. [42]
	*Sm1–7*	RT-EG	0.5 fg	gDNA, mice blood, serum	N/A	N/A	N/A	N/A	Song et al. [41]
	*28S-18S*	SGI/Gel	0.1 fg	gDNA, snails	N/A	N/A	N/A	N/A	Gandasegui et al. [35]
	*Sm1–7*	SGI/Gel	N/D	Urine	86	100% (81/81)	100% (5/5)	KK, PCR	Lodh et al. [49]
	*ITS*	SGI/Gel	70 fg	gDNA, snails	N/A	N/A	N/A	N/A	Caldeira et al. [38]
	*Mito*	SGI/Gel	N/D	Snails, stool	162	92.86% (12/13)	80.11% (112/149)	KK	Gandasegui et al. [39]
	*Sm1–7*	SGI/Gel	32 fg	gDNA, stool	383	97% (166/171)	100% (207/207)	KK	Mwangi et al. [43]
	*Sm1–7*	SGI/Gel		Urine	111	100% (97/111)	100% (14/14)	KK, PCR	Price et al. [51]
	*Mito*	RT-EG/SGI/Gel	0.01 fg/µL	Urine	28	71.4% (5/7)	71.4% (15/21)	KK	Fernández-Soto et al. [44]
	*Mito*	RT-EG/SGI/Gel	N/D	gDNA, hepatic, skin, appendix	N/A	N/A	N/A	N/A	García-Bernalt Diego et al. [52]
*Sj*	*SjR2*	SGI/Gel	0.08 fg	gDNA, serum	50	96.7% (29/30)	100% (20/20)	Micro, PCR	Xu et al. [45]
	*28S*	T/Gel	100 fg	gDNA, snails	N/A	N/A	N/A	N/A	Kumagi et al. [33]
	*SjR2*	Gel	0.1 fg	gDNA, rabbits blood	N/A	N/A	N/A	N/A	Wang et al. [47]
	*28S*	Calcein	100 fg	gDNA, snails	N/A	N/A	N/A	N/A	Tong et al. [36]
	*SjR2*	SG I	N/D	Human, rabbits serum	170	95.5% (105/110)	100% (60/60)	KK	Xu et al. [46]
	*28S*	Calcein	N/D	Snails	N/A	N/A	N/A	N/A	Qin et al. [40]

^1^ Species: *S* spp. (*Schistosoma* spp.) *Sh* (*S. haematobium*) *Sm* (*S. mansoni*) *Sj* (*S. japonicum*), ^2^ Mito: mitochondrial minisatellite. For all other abreviations see Figure 1, ^3^ SG I: SYBR Green I; Gel: Electrophoresis; T: Turbidity; RT-EG: Real-time EvaGreen fluorescence detection, ^4^ *Si: S. intercalatum; Sb: S. bovis,* ^5^ gDNA: parasite genomic DNA, ^6^ *n*: sample size, number of patients (when applicable, ^7^ Ref. diag: Technique used as diagnostic reference. Micro: Microscopy; KK: Kato-Katz technique, N/A: Non-applicable; N/D: Non-disclosed.

### 3.3. LAMP in Human Schistosomiasis Diagnosis

A number of studies have been conducted to evaluate the clinical application of LAMP in the diagnosis of human schistosomiasis. Different human samples have been tested and performance has been compared with various diagnostic methods. Two studies conducted by Xu et al. [45,46] have applied LAMP to serum samples from people infected with *S. japonicum* (determined by KK), living in endemic areas in Hunan Province, China. In a first study, to evaluate LAMP, 50 serum samples, including 30 *S. japonicum*-positive and 20 *S. japonicum*-negative controls from healthy individuals, were analysed and a 96.7% sensitivity compared to 60% by PCR was obtained [45]. Later, in a second study, 110 patient serum samples *S. japonicum*-positive were also analysed by LAMP and compared to ELISA and indirect hemagglutination assay (IHA) techniques. The sensitivity and specificity of LAMP resulted in 95.5% and 100%, respectively, whereas sensitivity and specificity of ELISA and IHA was 84.6% and 85.7%, and 91.8% and 88.1%, respectively [46]. These differences must be considered in the light of the different objectives of the diagnostic techniques in relation to the different clinical states of the disease. While serological techniques may indicate a past infection (or indirectly an active infection), molecular diagnostics target active infections (or at least residual DNA), thus being much more sensitive in acute states. In both studies, serum samples from people living in non-endemic areas of *S. japonicum* infections were used to determinate the high specificity obtained with LAMP. However, despite being people from non-endemic schistosomiasis areas, antibody detecting methods (ELISA and IHA) showed cross reactivity and lower specificity than LAMP assay [45,46]. On the other hand, 10/60 (16.7%) residents in endemic areas of schistosomiasis recognized as “healthy” people (*S. japonicum*-negative by KK, ELISA, and IHA) were diagnosed as LAMP-positive, suggesting that classical methods may lack sensitivity for low-intensity infections diagnosis [46]. Those results could also imply an imperfect specificity, although this reasoning is weakened by the lack of cross-reactivity in non-endemic subjects. LAMP has been also evaluated in the clinical determination of *S. mansoni* in stool [39] and urine samples [44,49,51], as well as *S. haematobium* in urine samples [48,49,50,53]. Regarding *S. mansoni* DNA detection in clinical stool samples, a first survey using the so-called SmMIT-LAMP was conducted in the low-transmission area of Umbuzeiro, Brazil [39]. Considering the parasitological findings by KK as reference, the SmMIT-LAMP resulted in an overall sensitivity of 92.86% and 80.11% specificity with a negative predicted value (NPV) of 99.33% but a scarce 26% positive predicted value (PPV). The percentage of false negative registered in this study can be partly explained with the greater sensitivity of SmMIT-LAMP over the classical KK technique, especially in those patients with a low egg-count in areas of low *S. mansoni* transmission [39]. In large-scale field trials, collecting stool samples to diagnose intestinal schistosomiasis can be extremely laborious and urine samples have been proposed as a good alternative as a source of cfDNA, due to their better handling and storage. In this sense, several studies have demonstrated the detection of *S. mansoni* cfDNA in human urine samples by different LAMP assays, including the one based on the 121 bp Sm1–7 repeated sequence in filtered urine samples field-collected in Ghana [49] and Zambia [51] and other using the SmMIT-LAMP in long-term frozen patients’ urine samples [52]. Consequently, the combination of the high efficiency of LAMP with urine samples could be suitable to use not only for well-equipped laboratories, but also for poor-resource laboratories in *S. mansoni*-endemic areas. Additionally, SmMIT-LAMP was also successfully used on a skin biopsy as a real-time LAMP assay to confirm ectopic cutaneous schistosomiasis caused by *S. mansoni*, a proof of concept of the usefulness of LAMP in acute schistosomiasis diagnosis [54].

The microscopic detection of excreted ova in urine samples remains the ‘gold standard’ diagnostic method for *S. haematobium* infection despite its low sensitivity, high day-to-day variability and inefficacy in the acute stage of the disease. With the aim to solve this, a LAMP assay specifically designed for the detection of *S. haematobium* in patients’ urine samples was first developed by Gandasegui et al. in 2015. Compared to microscopy (69.2%), LAMP resulted in a higher sensitivity (86.7%); moreover, the simple heating of urinary pellets for DNA purification (the Rapid-Heat LAMPellet method) was effective to detect *S. haematobium* through LAMP in several urine samples with confirmed infection [48]. Later, this LAMP procedure was applied under field conditions using both purified DNA and heat-treated urine samples in comparison with microscopy in 172 human urine samples collected in a schistosomiasis-endemic area in Cubal, Angola [50]. The overall prevalence detected by LAMP was significantly higher than microscopy when testing purified DNA (73.8% vs. 50.6%), even when testing crude urine samples (63.4% vs. 50.6%). Nevertheless, the reproducibility of LAMP tests in a well-equipped laboratory only reached 72.1% and 49.5% of coincidences in DNA or crude urine, respectively. Test performance, especially in crude urine samples (usually containing many inhibitors which may interfere in DNA amplification), was probably affected by inappropriate sample storage (suffering from numerous freezing and thawing cycles) resulting in deterioration of DNA over time [50]. In a study conducted by Lodh et al. [49] in a schistosomiasis-endemic area of Ghana, LAMP was also used to detect *S. haematobium* in urine samples using two different DNA extraction methods, standard extraction kit and field usable LAMP-PURE kit. Urine samples were collected from 86 individuals with no previous parasitological examination for *S. haematobium* and were evaluated by LAMP and PCR. LAMP amplification for both extractions showed similar sensitivity (72/86; 84%) when compared with PCR (70/86; 81%) showing that LAMP for detecting *S. haematobium*-specific DNA is an effective diagnostic tool, equal to PCR amplification [49]. Another study evaluated a LAMP method based on ribosomal IGS DNA to detect *S. haematobium* in 69 urine samples collected from suspected patients for urogenital schistosomiasis attending outpatient clinic in Imbaba Cairo, Egypt. LAMP resulted in a 100% sensitivity and 63.16% specificity when compared with conventional urine filtration followed by microscopical egg detection [53].

### 3.4. LAMP to Evaluate Treatment Success

**Figure 2:**
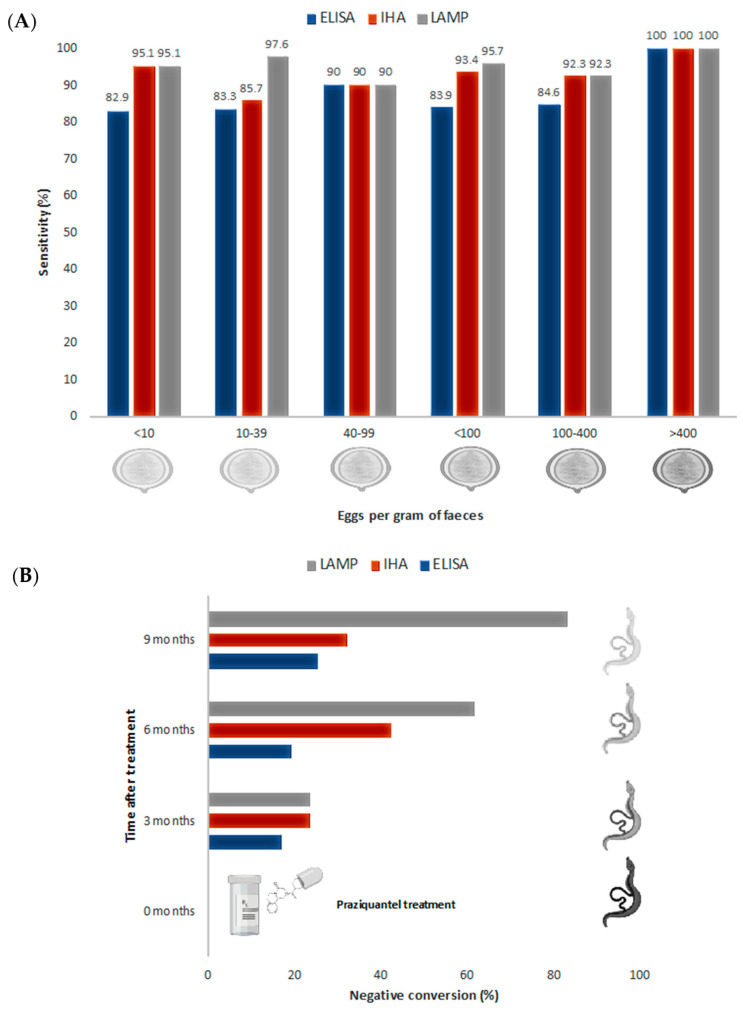
Sensitivity and post-treatment evaluation comparison for *S. japonicum* experimental infection by ELISA, IHA and LAMP. (**A**) ELISA, IHA and LAMP sensitivity comparison, related to the number of eggs per gram of faeces. (**B**) Evaluation of negative conversion rates after praziquantel treatment by ELISA, IHA and LAMP. Data obtained from Xu et al. [46].

The evaluation of schistosomiasis treatment success with diagnostic tools has been traditionally very limited, due to the low sensitivity of parasitological methods, particularly in light infections, and the limited value of antibody-based immunological methods. On the other hand, serum-PCR methods have shown their usefulness to evaluate treatment success on a long-term basis (1-year after initial treatment), not in early treatment monitoring [55]. In this sense, LAMP assays have been used to evaluate the efficacy of chemotherapy in schistosomiasis. However, studies are still limited to *S. japonicum* infections, performed both in experimentally infected animals [45,46,47] and in patients [46]. In a first study, Xu et al. [45] studied praziquantel treatment in rabbits experimentally infected with a high dose of *S. japonicum* (500 cercariae). They showed that LAMP become negative after 20 weeks post-infection (12-week post-treatment), two weeks longer than with PCR. Subsequently, Wang et al. [47] tested the value of LAMP to evaluate treatment success in lighter *S. japonicum* experimentally infected (200 cercariae) rabbits. In this case, both artesunate and praziquantel treatment were evaluated. Again, LAMP showed a higher sensitivity than PCR, detecting *S. japonicum* DNA in rabbit sera up to 20 weeks post-treatment in 50% and 66% of the cases, treated with artesunate and praziquantel, respectively. PCR was able to detect *S. japonicum* DNA only up to 12 and 8 weeks post-treatment with artesunate and praziquantel, respectively [47]. Finally, in an extension of their previous work, Xu et al. [46] also evaluated LAMP effectiveness for the detection of light infections in experimentally infected rabbits pre- and post-treatment and also in chemotherapy efficacy in human patients. On the one hand, rabbits were infected with 30 *S. japonicum* cercariae and subsequently treated with praziquantel. LAMP could detect *S. japonicum* DNA in sera from infected rabbits at the third day post-infection and became negative at 10 weeks post-therapy, showing the usefulness of LAMP in early diagnosis of light infections and also in treatment success evaluation. ELISA and IHA techniques were used to compare and assess LAMP results. As expected, they were not useful for early diagnosis (ELISA and IHA gave positive results at 5 and 4-weeks post-infection, respectively) and, anti-schistosome antibodies were detected by both techniques during 23 weeks after treatment [46]. On the other hand, treatment efficacy was evaluated in 47 patients’ sera infected with *S. japonicum*. The parasite DNA in serum was not detected in 31.9%, 61.7% and 83% of patients at 3 months, 6 months and 9 months post-therapy, respectively. LAMP negative conversion rates were higher than those of IHA and ELISA, that reached only 31.9% and 25.5%, respectively, at 9 months post-treatment (Figure 2). These results seem to indicate that LAMP technique has potential for monitoring the effectiveness of schistosomiasis treatment in a long-term approach, not only in early treatment monitoring. However, further studies are needed to determine the usefulness of LAMP in assessing the efficacy of treatment. In addition, the role of free DNA in biological samples, as well as our capacity to link DNA detection levels to biological entities (i.e., eggs or worms) is still challenging for many parasites [56] and needs to be clearly addressed to give real meaning to the results obtained. Specifically, for *Schistosoma* spp. infections, three different hypotheses could explain the mentioned results. Firstly, the subcurative doses of praziquantel regularly used, that would generate persistence infection in some patients. Secondly, DNA derived from degenerating eggs trapped in tissues could be slowly being released, thus, yielding false-positive results regarding active infections. Finally, single-sex *Schistosoma* infections have been described. Those infections could be producing positive results without the excretion of eggs [55].

### 3.5. Towards a True Point-of-Care Schistosomiasis Diagnostic?

Accurate patient identification is still a major limitation in NTDs management and control, dramatically contributing to the sustained burden that many of these diseases still present [57]. Molecular tools, highly sensitive and precise methodologies, have not yet replaced microscopy or serology in schistosomiasis diagnosis despite repeatedly showing better results at the laboratory. This is partly caused by the difficulty of deploying molecular methods to the field. Such methodologies should fulfill the rules established by the acronym ASSURED (Affordable, Sensitive, Specific, User-friendly, Rapid and Robust, Equipment-free, Deliverable) a set of criteria that must be achieved for any diagnostic method to be considered a point-of-care (POC) test [58]. The term has been recently revisited and modernized to REASSURED by Land et al. [59], including: Real-time connectivity and Ease of specimen collection and Environmental friendliness. In this direction, several studies show promising results contributing to develop accurate and specific schistosomiasis POC diagnosis.

To date, nucleic acid purification is considered the most important challenge preventing molecular diagnostics adoption from reaching the field [60]. In this sense, *S. haematobium* detection in clinical urine samples with LAMP has been accomplished with a simplified DNA extraction consisting on a 15-min 95 °C lysis step (so-called rapid-heat LAMPellet method) [34,49]. The ability of LAMP to amplify DNA without prior extraction has been proved in a wide variety of body fluids (i.e., plasma, blood, urine, saliva or semen) [61]. This has allowed the use of alternative specimens, such as urine samples, for the molecular diagnosis of intestinal schistosomiasis [44,49,51]. As handling and storage of large numbers of stool samples for field surveys is very demanding, urine has been proposed as more suitable specimen for large-scale field studies [44,62]. Nevertheless, further evidence is needed to prove the usefulness of urine for intestinal schistosomiasis diagnosis. As nucleic acid purification can be avoided and detection of LAMP results can be done with economical dyes [22,23], a considerable reduction in the final cost is achieved. LAMP costs are estimated to be 0.71–2$ per sample while for PCR it is 6.4–7.7$, ELISA is 1.5$, and KK is 2.00–2.67$ [63]. However, a bias can be attributed to those estimations as DNA purification is not included and, to date, rapid extraction methods reproducibility has not been proved in schistosomiasis large-scale surveys.

Another interesting approach to develop POC diagnostic for schistosomiasis is the use of ready-to-use formats, stabilized reaction mixes for LAMP that permit avoiding cold chain maintenance and facilitate the performance of the diagnosis by untrained personnel. Our group has recently presented a novel protocol for long-term preservation of LAMP master mixes for *S. mansoni* detection. We developed a simple one-step protocol based on threhalose as cryoprotectan to produce functional ready-to-use reaction mixes in at least 3 weeks or over five months when storing at room temperature or at 4 °C, respectively [52]. Other dry-LAMP approaches for schistosomiasis based on distinct cryoprotectans (i.e., sucrose) have been reported too [37], thus potentially allowing them to work at room temperature and reducing equipment needed in field settings. Despite all recent advances, no significant changes in current diagnostic protocols for schistosomiasis have included LAMP method. This technology has been available since 2000 [16] but it is not yet a true POC test, nor are any other PCR-based techniques.

It should also be noted that LAMP presents a several important disadvantages: it is non-applicable for cloning, it has a highly constrained primer design, the risk of carry-over contamination is elevated, and multiplexing approaches are still scarce and mechanistically, very complex [64]. In addition, partial hybridization of one or more LAMP primers to fragmented genomic host DNA or with phylogenetically related non-target microorganisms abundant in non-sterile biological materials may yield occasionally random amplification by the *Bst* polymerase used for LAMP. The difficulty to develop multiplex approaches is the most concerning out of the limitations described in the case of schistosomiasis, since co-infections are very frequent in endemic regions, adding one more layer of complexity to an already difficult diagnosis. However, some multiplex-LAMP (mLAMP) approaches are beginning to appear for detection of a number of parasites causing infection diseases and a two-stage isothermal amplification method has been applied to schistosomiasis. Briefly, a microfluidic chip has been designed to perform a dubbed rapid amplification (RAMP) first-stage follow by a second-stage LAMP assay. This assay has been designed in a 16-plex, 2-stage RAMP assay to simultaneously detect up to 16 different targets of DNA and RNA from different parasites in just 40 min, including *S. mansoni, S. hematobium* and *S. japonicum* [65].

Finally, although LAMP presents sensitivity and specificity features to be a standalone diagnostic technique, the combination with other useful diagnostic tests should also be considered. This is especially true for the widely used detection of circulating cathodic antigen (CCA) and circulating anodic antigen (CAA). Both have already been developed adapted to lateral flow dipsticks, specifically CAA for *S. japonicum* and CCA for *S. mansoni*. Still, CCA antigen presents limitations detecting *S. haematobium*, thus is only considered effective in regions where only *S. mansoni* infections occur [14]. Thus, LAMP would be a great complementary diagnostic tool in those regions where infections by both *S. mansoni* and *S. haematobium* occur.

On the basis of the REASSURED criteria [59] defined above, LAMP already fulfills most of the requirements. It is affordable, sensitive, specific, rapid and robust by defi-nition, and efforts have been made to develop supportive technology to make it more user friendly. Nevertheless, some others still need improvement. The ease of specimen collec-tion is still hampered by the lack of validation of alternative specimens (i.e., urine for *S. mansoni* diagnostic) in large scale-studies, as well as the true capability of rapid and equipment-free DNA purification strategies. Additionally, it is very unlikely that LAMP becomes a completely equipment free technology, but it can be reduced and simplified to the fullest (through lateral flow dipsticks, microchips and other lab-on-chip displays) and, critically, made more affordable. This, combined with the extension of smartphone tech-nology in Sub-Saharan Africa, and the development of smartphone diagnostic strategies [66], could finally bridge the gap of real-time connectivity and data management in far remote areas. However, real world application of this technology has been coming for years now, and it has not yet been delivered to those who needed most. Here, the extensive research made and the steps needed ahead have been highlighted. Still, the sustained invariability on the diagnostic tools used in tropical diseases to date begs the question: Is it the Research or is it the Health Systems?

## 4. Conclusions

Schistosomiasis still represents a very pressing health problem in many countries of the world. There is a critical need for new specific and sensitive diagnostic tools that are inexpensive and easy to transport and use. Although being one of the NTDs for which more work has been done, clinical applications of LAMP for schistosomiasis diagnosis are still limited and only applied in research scenarios, not routinely in the clinic. Larger, in-depth studies are imperative to move forward in the real-world application of this method. We believe that with the help of new supportive technology (i.e., including lateral flow disks, microchips, lab-on-chips, smartphone apps) LAMP might be able to finally reach endemic areas. LAMP results obtained for schistosomiasis have proved to be comparable to or better than those of the common diagnostic methods and it could be a great candidate to finally get molecular testing to the field as a true POC test.
